# Machine Learning-Based Prediction of Performance Gaps in Rowing and Identification of Key Training Monitoring Indicators

**DOI:** 10.3390/s26103006

**Published:** 2026-05-10

**Authors:** Jianyu Li, Guochun Liu, Wenjin Wang, Chunmei Cao

**Affiliations:** 1 Division of Sports Science and Physical Education, Tsinghua University, Beijing 100084, China; m18844093378@163.com (J.L.); lgc890206@163.com (G.L.); wangwenjin0213@163.com (W.W.); 2 College of Exercise Medicine, Chongqing Medical University, Chongqing 400331, China

**Keywords:** machine learning, rowing, sensor-derived monitoring, biomechanical monitoring, decision support

## Abstract

Although routine biomechanical monitoring in rowing increasingly relies on sensor-based and instrumented measurement systems that can capture multidimensional performance indicators with considerable precision, systematic approaches are still needed to integrate these sensor-derived data into a unified monitoring dataset and translate them into decision support for practice. This study aimed to construct a unified rowing training monitoring dataset from real-world sensor-derived biomechanical measurements, develop predictive models for athletes’ performance gaps relative to target 2 km performance, and, for target attainment classification, identify key training monitoring indicators and evaluate their practical value in training practice. A total of 249 biomechanical testing records collected during the 2024–2025 season from the Chinese National Rowing Team were included. After standardized processing, 449 athlete-level records were generated for the primary analysis. Following exclusion of observations with missing primary regression labels, 172 modeling records were retained, corresponding to 87 test reports and 119 athletes. The primary regression outcome was the percentage time difference relative to target 2 km performance. XGBoost Regressor, Elastic Net, and LASSO were used for regression modeling, whereas Logistic Regression and XGBoost Classifier were used for the secondary classification task of target attainment. Internal validation was performed using grouped cross-validation at the athlete level, and model interpretation was supported by permutation importance, sparse linear coefficients, and robustness analyses. The results showed that all formal models outperformed their respective baseline models. In the primary regression task, XGBoost Regressor achieved the best performance in terms of MAE, whereas Elastic Net performed best in RMSE and R^2^. The key training-monitoring indicators mainly included mean boat velocity, minimum boat velocity, stroke rate, distance per stroke, and efficiency-related variables. After removal of grouping variables related to boat class, sex, and weight category, the performance of XGBoost Regressor remained largely stable, suggesting that the primary predictive signal was mainly derived from measured technical and biomechanical features. In the secondary classification task, XGBoost Classifier achieved an ROC AUC of 0.992. This study provides a team-specific applied framework for extending sensor-derived rowing monitoring outputs from multi-indicator measurement toward interpretable performance evaluation and decision support within an elite-team setting, while broader external validation remains necessary.

## 1. Introduction

Rowing is a sport in which endurance capacity and technical execution are highly coupled, and performance is therefore not determined by any single physical or technical indicator, but rather by the combined influence of boat-speed regulation, stroke-rate organization, propulsive efficiency, movement timing and crew coordination in multi-athlete boats [1]. With the continued development of onboard instrumentation and sensor technologies, variables such as boat velocity, stroke rate, acceleration, and oar blade dynamics can now be continuously captured and monitored in real-world training and competition settings with increasing precision [2]. Recent work has shown that sensor technologies and instrumented monitoring systems have enabled increasingly detailed assessment of rowing biomechanics in both training and competition settings [3]. However, technical evaluation in practice still relies heavily on experiential judgment, while existing research has focused largely on linear associations between isolated indicators and performance [4]. Consequently, systematic evidence remains limited regarding how multi-source training monitoring data can be integrated to identify key determinants and to support sport-specific performance evaluation and training decision making [5].

In recent years, artificial intelligence, particularly machine learning, has been increasingly applied in competitive sport across areas including performance analysis, training-load evaluation, injury risk prediction, and individualized intervention [6,7,8]. Relative to traditional statistical approaches, its key advantage lies in the ability to detect latent patterns within high-dimensional, complex, and interdependent data, thereby improving the characterization and prediction of sport-specific performance [9,10]. In rowing, where performance is jointly shaped by multiple interacting factors, machine learning offers not only a means of integrating otherwise discrete monitoring indicators and improving analytical efficiency, but also a new data-driven pathway for identifying key technical characteristics [11,12]. In high-performance sport, however, the practical value of such models depends not only on predictive accuracy, but also on whether their outputs are interpretable, can be meaningfully reconciled with sport-specific training knowledge, and can be translated into actionable feedback for coaching and training practice [13].

On this basis, the present study drew on real-world training monitoring data from the Chinese National Rowing Team to establish a unified analytical framework integrating boat-level, individual technical, and evaluation-level information. Using the percentage time difference relative to target 2 km performance as the primary outcome [14], we developed models to predict performance gaps and further identified key training-monitoring indicators associated with those gaps. The study was intended to provide more objective, precise and interpretable data support for routine rowing monitoring, and specifically to address how sensor-derived rowing monitoring data can be translated into interpretable decision support for training evaluation, performance limitation identification, and intervention prioritization.

## 2. Materials and Methods

### 2.1. Study Design

This study was an applied modeling study conducted in a real-world training setting within the Chinese National Rowing Team. The study sample comprised empirical data derived from 2 km biomechanical tests completed by national team athletes during the preparation phases of the 2024 and 2025 seasons. The aim was to construct a high-quality training monitoring dataset from longitudinally tracked sport-specific testing results and, based on this dataset, to develop models for performance gap prediction and target-attainment classification, thereby identifying the training monitoring indicators most closely associated with sport-specific performance gaps and evaluating their potential practical value in athletic training. This study did not rely on a pre-existing public benchmark dataset; rather, it retrospectively analyzed empirical measurements generated during routine elite-rowing monitoring. To improve transparency and reproducibility, the de-identified analytical dataset used for the present manuscript is provided in the Supplementary Materials. This study was approved by the Institutional Review Board of Tsinghua University (Approval No. 20180016). All procedures were conducted in accordance with the Declaration of Helsinki. Written informed consent was obtained from all participants prior to inclusion in the study. Accordingly, the present study should be interpreted as a team-specific applied modeling study based on one elite rowing monitoring environment, rather than as a broadly validated general methodology.

### 2.2. Data Sources and Development of the Training Monitoring Dataset

The study data were not obtained from a public or readily available repository. Instead, they were retrospectively derived from routine sensor-based and instrumented biomechanical monitoring of athletes from the Chinese National Rowing Team. These measurements represented real-world monitoring outputs from elite-rowing practice and covered multiple dimensions, including boat-level, individual technical, and target evaluation information. The analyzed variables therefore reflected sensor- and instrumentation-derived biomechanical signals, including boat velocity, stroke rate, boat acceleration, oar-blade efficiency, drive time, rhythm, catch angle, finish angle, total angle, slip, effective angle, force output, work per stroke, and related performance indicators.

The monitored variables used in the present study were generated during routine on-water biomechanical testing conducted within the Chinese National Rowing Team. The original testing reports indicate that the monitoring outputs were generated within a BioRow-based biomechanical testing and analysis workflow (BioRow Ltd., Slough, UK) and included structured boat-level, individual technical, force-related, angle-related, and selected body segment kinematic variables. Accordingly, the analytical dataset used in the present study was derived from exported monitoring outputs and structured derived variables, rather than from raw continuous sensor streams directly modeled from first acquisition.

Data export, cleaning, and subsequent dataset construction were performed retrospectively from routine team-monitoring records. Before analytical dataset construction, the exported records were checked for consistency, standardized, and matched across levels according to the study protocol described below. Because the present study relied on retrospective analytical files generated in a real-world monitoring setting, complete hardware model identifiers, raw acquisition settings, and detailed calibration records were not available for full reconstruction in the current manuscript.

To construct a unified training monitoring dataset, the longitudinally tracked empirical variables were first standardized and organized into a structured data table, including information at the test session level, athlete level, boat-sample level, individual technical-sample level, and target evaluation level. Subsequently, the evaluation stroke rate from each athlete’s single test session was defined as the primary analytical unit, and was matched to the nearest boat-level sample and individual technical sample. Matching was performed within the same test session and based on nearest-neighbor rules using the smallest differences in time and stroke rate between the evaluation stroke rate and the corresponding boat-level and individual technical samples. When multiple candidate samples were available, the one with the minimal difference was retained. This process yielded the training-monitoring dataset used for the primary model analyses. The included data covered testing results across different boat classes, including single sculls, pairs/doubles, fours/quads, and eights, as well as different rowing styles (sweep and sculling), and athletes of different sexes and weight categories. To preserve comparability within a unified training monitoring framework, grouping variables such as boat class, crew size, rowing style, sex, and weight category were retained during dataset construction and were subsequently controlled for and examined in later modeling and sensitivity analyses. For transparency and reproducibility, the de-identified analytical dataset used for the present manuscript-level analyses is provided in the Supplementary Materials. The shared dataset corresponds to the structured analytical file after variable standardization, matching, and de-identification, rather than to the raw source monitoring files generated during routine team operations.

### 2.3. Outcome Definition

The primary outcome was defined as the performance gap relative to the target, rather than the absolute performance result. The primary regression label was the percentage time difference between the athlete’s current test result and the target 2 km performance, which was used to quantify the degree of deviation between current technical status and the predefined sport-specific performance target. Relative to absolute time, this outcome more directly captures performance status with respect to the target within the current training evaluation framework and, to some extent, reduces the influence of baseline differences in absolute performance across event contexts. A binary target attainment task was subsequently derived as a secondary classification analysis. In the primary classification scheme, target attainment was defined according to whether the percentage time difference met the 0% threshold, while 1% and 2% thresholds were additionally examined in sensitivity analyses. Because the classification label was derived directly from thresholding the regression label, classification was treated as a secondary rather than primary analysis. Stability of the classification results with respect to threshold specification was further evaluated using alternative threshold schemes.

### 2.4. Feature Selection and Leakage Control

**Figure 1:**
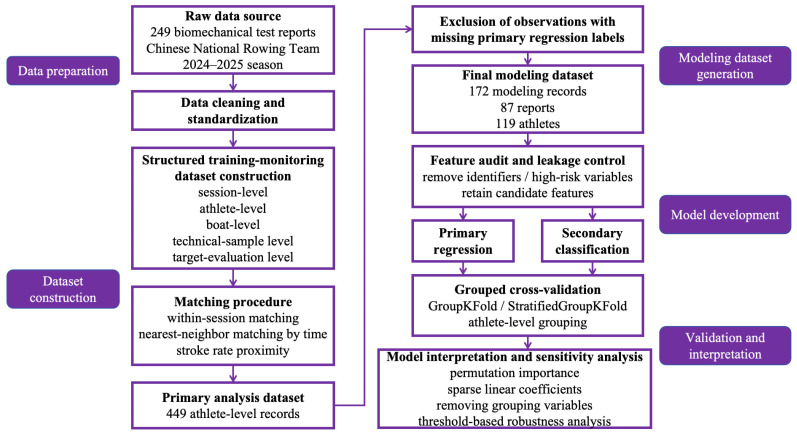
Overall study workflow of dataset construction, modeling, validation, and interpretation.

To ensure methodological rigor and result interpretability, candidate variables were systematically audited and hierarchically managed prior to model development. According to their source, functional attributes, and potential modeling roles, variables were classified as identifier variables, grouping variables, boat-level features, individual technical features, metadata features, or potentially high-risk variables. The primary model feature set comprised boat-level mechanical indicators, individual technical and biomechanical variables, and a limited number of stable metadata features, whereas identifier variables, raw tracking fields, and evaluation-derived fields directly sharing the same origin as the outcome were excluded. Variables with potential information-leakage risk were explicitly removed, resulting in 46 retained candidate features for the primary regression and classification analyses [15,16]. Grouping variables, including boat class, crew size, rowing style, sex, and weight category, were retained as covariates in the primary models. Sensitivity models excluding these grouping variables were then constructed to examine whether model performance was primarily driven by sport-specific technical, and biomechanical features, thereby improving the robustness of interpretation and the credibility of the conclusions. The overall study workflow is illustrated in Figure 1.

### 2.5. Principles and Comparative Rationale of the Machine Learning Methods

To balance predictive performance and practical interpretability, the present study included both linear and nonlinear models, rather than relying on a single algorithm. This design allowed comparison across three complementary dimensions: formal models versus naïve baselines, linear versus nonlinear methods, and prediction accuracy versus interpretability.

For the primary regression task, Elastic Net, LASSO and XGBoost Regressor were included. Elastic Net was selected because it combines coefficient shrinkage with variable selection and is suitable for correlated predictors, which was relevant given the biomechanical relatedness among many monitoring variables. LASSO was included as a sparser linear method to help identify a more compact set of core predictors. These two models therefore served not only as predictive models but also as interpretable linear references. XGBoost Regressor was included because rowing performance gaps are likely to reflect nonlinear relationships and interactions among boat speed, rhythm, efficiency, and technical variables that may not be fully captured by linear models.

For the secondary classification task, Logistic Regression and XGBoost Classifier were included. Logistic regression served as an interpretable linear benchmark, whereas XGBoost Classifier was used to examine whether nonlinear structure in the monitoring variables improved target attainment discrimination. In addition, baseline models were used in both tasks to determine whether the formal models provided predictive value beyond trivial reference estimates.

Overall, the purpose of this comparative design was not simply to identify the single best-performing algorithm, but to assess whether different model classes converged on meaningful training monitoring signals and whether predictive modeling could be aligned with interpretable support for rowing training evaluation and decision making.

### 2.6. Machine Learning Modeling Workflow, Cross-Validation, and Model Evaluation

Regression and classification models were developed within a unified framework of data preprocessing and grouped cross-validation. For the regression task, Elastic Net, LASSO, and XGBoost Regressor were included, with a baseline model used as a reference. For the classification task, Logistic Regression and XGBoost Classifier were included, together with corresponding baseline classifiers for comparison [17,18,19]. Considering the sample size, model complexity and the applied focus of the study, large-scale automated hyperparameter tuning and nested cross-validation were not performed. Instead, each model was compared across a predefined and limited set of candidate hyperparameter combinations using grouped 5-fold cross-validation [20], and the best-performing parameter set was selected accordingly. Model selection followed prespecified rules: for the regression task, the primary selection criterion was the lowest mean absolute error (MAE). In the case of ties, root mean square error (RMSE) and then the coefficient of determination (R^2^) were used sequentially as tie breakers. For the classification task, the primary selection criterion was the highest area under the receiver operating characteristic curve (ROC AUC). In the case of ties, mean balanced accuracy and then mean F1 were used sequentially as tie breakers.

All models were evaluated using grouped cross-validation. GroupKFold (k = 5) was used for the regression task, whereas Stratified GroupKFold (k = 5) was preferentially used for the classification task [20,21]. If the data structure did not satisfy the relevant requirements, GroupKFold was used as a fallback. The grouping key was defined by modeling_split_group, ensuring independence between the training and validation sets at the athlete level. Given the modest effective modeling sample for the primary task relative to the dimensionality of the candidate predictor space, the modeling strategy was intentionally kept conservative. Accordingly, large-scale automated hyperparameter tuning and nested cross-validation were not performed, and model comparison was restricted to a limited set of prespecified candidate parameter combinations under athlete-level grouped internal validation.

All preprocessing steps were embedded within the Pipeline and Column Transformer and executed entirely within the cross-validation workflow to prevent information leakage arising from preprocessing on the full dataset. For linear models, numerical features were imputed using the median and subsequently standardized. For tree-based models, numerical features were imputed using the median without standardization. Low-cardinality categorical variables were encoded using one hot encoding. All preprocessing parameters were estimated within each training fold and applied only to the corresponding validation fold.

Model performance in the primary regression task was evaluated using mean absolute error, root mean square error, and the R^2^, whereas the primary classification task was evaluated using the ROC AUC, F1 score, balanced accuracy, and the Brier score [22,23,24]. In addition to fold-wise evaluation, out-of-fold predictions were generated to support visualization of observed-versus-predicted scatter plots and residual distributions for the regression task, as well as calibration curves, ROC curves, and precision recall curves for the classification task. For the regression models, MAE, RMSE, and R^2^ were jointly compared across candidate models, and the best MAE model and the best interpretable linear benchmark model were identified separately.

### 2.7. Model Interpretation and Sensitivity Analyses

To improve the credibility of model interpretation and its relevance to training practice, we combined global importance analysis with sparse linear feature signatures. For the tree-based models, permutation importance was used to evaluate the relative contribution of each candidate variable to predictive performance, thereby identifying the key training-monitoring indicators most closely associated with the performance gap relative to the target [25]. Because this approach does not depend on a model-specific internal parameter interpretation framework, it provides a relatively robust global ranking of variable importance. For Elastic Net and LASSO, non-zero coefficients, together with their directional information, were further extracted to construct sparse linear feature signatures, allowing identification of core variables that retained stable contributions within a more parsimonious linear framework and facilitating interpretation of the potential directional roles of key indicators identified in the primary models. On this basis, multilevel sensitivity analyses were further conducted, including technical-feature models that excluded grouping variables, in order to examine whether primary model performance was driven mainly by sport-specific technical and biomechanical features rather than by background labels such as boat class, rowing style, sex, or weight category; in addition, alternative threshold schemes were evaluated for the target-attainment classification task to assess the stability of classification performance with respect to threshold specification.

### 2.8. Statistical Software and Implementation Environment

All data processing, construction of the training-monitoring dataset, model development, and result visualization were conducted in Python 3.13.5. The main libraries used included pandas 2.3.3, NumPy 2.3.5, scikit-learn 1.7.2, XGBoost 3.1.2, matplotlib 3.10.7, SciPy 1.16.3, and SHAP 0.51.0. All analytical procedures were implemented on the basis of a unified frozen dataset and a prespecified feature plan to ensure reproducibility of the results and consistency of the analytical workflow.

## 3. Results

### 3.1. Sample and Dataset Characteristics

A total of 249 biomechanical test reports collected during the 2024–2025 training seasons of the Chinese National Rowing Team were included in the study, and all records were successfully processed through data cleaning, standardization, and structured organization. On this basis, 449 athlete-level records were generated for the primary analysis, retaining multi-level empirical information including athlete characteristics, boat-level samples, individual technical samples, evaluation-level data, and segmented biomechanical variables. This dataset provided relatively comprehensive coverage of the key biomechanical indicators routinely obtained in training monitoring and supported the subsequent analyses of performance gap prediction, target attainment classification, and identification of key monitoring indicators within a real-world training context.

The primary analytical dataset covered six boat classes, including single sculls, pairs, doubles, fours, quads, and eights, and also included both sweep and sculling events as well as test results from athletes of different sexes and weight categories. Overall, the sample was dominated by two-person and four-person boat classes. After restricting the dataset to observations with non-missing primary regression labels, 172 modeling records were retained, corresponding to 87 test reports and 119 athletes. This reduction from the broader primary analysis dataset was driven by the requirement for complete primary regression labels in the main modeling task, rather than by post hoc sample trimming. To avoid introducing additional uncertainty into the primary outcome, observations with missing primary regression labels were not imputed and were excluded from the main regression analysis. The modeling sample was drawn primarily from two-person and four-person boats and was further characterized by a predominance of sweep events, male athletes, and open weight categories. The primary regression label was complete across all primary modeling sub-cohorts, indicating good overall completeness of the final modeling sample with respect to both observational boundaries and label availability.

### 3.2. Primary Regression Results

**Figure 2:**
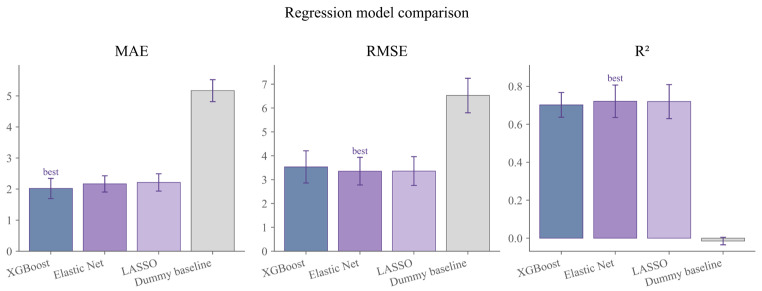
Comparison of predictive performance across primary regression models under grouped cross-validation.

In the analysis using the percentage time difference relative to target 2 km biomechanical test performance as the primary outcome, all formal models substantially outperformed the baseline model (Figure 2). Based on grouped cross-validation, XGBoost Regressor achieved the best performance in terms of mean absolute error, with an MAE of 2.021, whereas Elastic Net performed best in terms of root mean square error and coefficient of determination, with an RMSE of 3.356 and an R^2^ of 0.721. By comparison, the baseline model yielded an MAE of 5.169, an RMSE of 6.524, and an R^2^ of −0.015, indicating that models derived from training-monitoring indicators were able to capture athletes’ performance gaps relative to the target more effectively than a crude mean-based estimate.

Some divergence in performance was observed across models depending on the evaluation metric. XGBoost Regressor showed the strongest performance in controlling absolute prediction error and was therefore considered the best-performing model with respect to MAE. Elastic Net, by contrast, demonstrated better overall fitting stability while retaining stronger interpretability, and was therefore regarded as the best interpretable linear benchmark model in the present study. The overall performance of LASSO was similar to that of Elastic Net, but it was slightly inferior across all three metrics, including MAE, RMSE, and R^2^. On this basis, model performance was evaluated from two complementary perspectives, namely predictive performance and interpretability.

**Figure 3:**
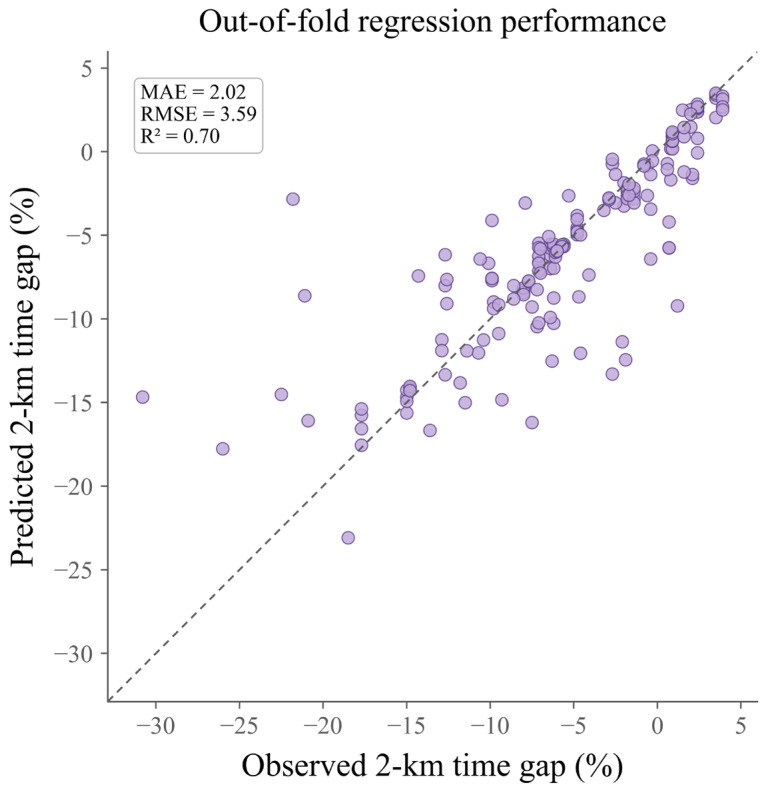
Out-of-fold predictive performance of the best primary regression model. Each purple point represents one observation from the out-of-fold predictions, and the dashed diagonal line indicates the line of identity between observed and predicted values.

Further inspection of the regression scatter plot showed that the predicted values were generally distributed around the line of identity, suggesting that the best MAE model captured the overall variation in performance gaps relative to the target reasonably well (Figure 3). Although a small number of observations deviated from this pattern in the range of larger negative gaps, the overall fit remained clear. Taken together, the model comparison results and out-of-fold prediction patterns suggest that, within the present grouped internal validation setting, XGBoost Regressor was more suitable as the primary performance model, whereas Elastic Net provided a more appropriate linear reference for subsequent identification of key monitoring indicators and interpretation of model behavior. These results should nevertheless be interpreted with caution given the modest effective modeling sample of the primary task.

### 3.3. Identification of Key Training Monitoring Indicators and Model Interpretation

**Figure 4:**
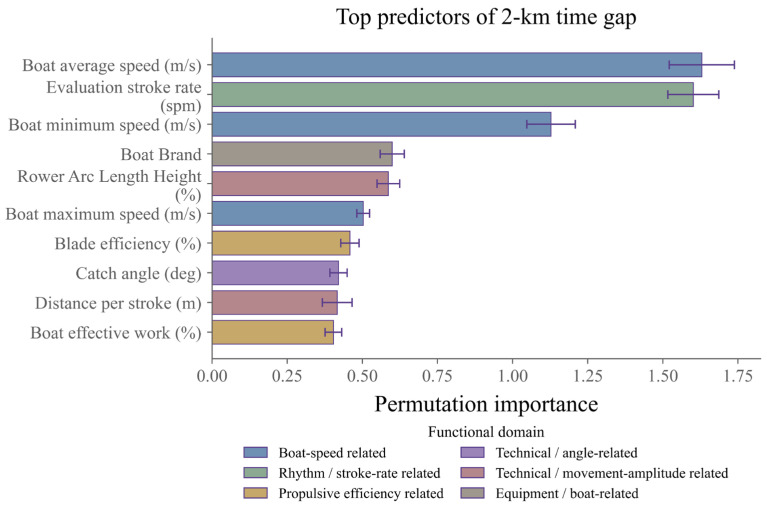
Global importance ranking of key training-monitoring indicators in the primary regression model.

Model interpretation indicated that the key training-monitoring indicators identified by the primary regression models were primarily related to boat-speed strategy, stroke-rate regulation, propulsive efficiency, and individual technical characteristics (Figure 4). Based on the global permutation-importance ranking, mean boat velocity, stroke rate, and minimum boat velocity emerged as the three most important predictors, with importance values of 1.6294, 1.6014, and 1.1279, respectively. They were followed by arc length relative to height, maximum boat velocity, oar-blade efficiency, catch angle, distance per stroke, and effective power, suggesting that the models were particularly sensitive to boat kinematics, technical-rhythm variables, and indicators of movement amplitude and efficiency. The markedly greater importance of mean boat velocity, stroke rate, and minimum boat velocity further indicates that boat-speed maintenance and stroke-rhythm organization were central to explaining variation in performance gaps relative to the target during the 2 km biomechanical test.

Results from the sparse linear models further demonstrated substantial agreement between Elastic Net and LASSO in core variable selection (Table 1). Both models retained maximum boat velocity, evaluation stroke rate, stroke rate, distance per stroke, minimum boat velocity, mean boat velocity, and boat efficiency in the core sparse feature set, suggesting that these variables not only ranked highly in the global importance analysis of the nonlinear model but also contributed stably within a more parsimonious linear framework. In the Elastic Net, the largest absolute coefficients were concentrated in boat speed, rhythm control, power output, and propulsive efficiency variables, and the corresponding coefficient directions in the LASSO model were broadly consistent with the overall pattern. LASSO additionally retained individual technical indicators such as catch slip. Overall, the primary regression model appeared to rely not on a single dominant variable, but on a composite feature structure jointly defined by boat-speed level, technical rhythm, and propulsion efficiency related indicators.

### 3.4. Robustness Analyses and Secondary Classification Secondary

Robustness analyses of the primary regression models showed that, after removal of grouping variables including boat class, crew size, rowing style, sex, and weight category, the predictive performance of XGBoost Regressor changed only marginally, with MAE increasing slightly from 2.021 to 2.027, RMSE decreasing from 3.534 to 3.436, and R^2^ increasing from 0.702 to 0.716. By contrast, the performance of Elastic Net deteriorated more noticeably, as reflected by an increase in MAE from 2.168 to 2.621, an increase in RMSE from 3.356 to 3.678, and a decrease in R^2^ from 0.721 to 0.678. These findings suggest that the primary predictive signal of the best MAE model did not depend primarily on background grouping variables such as boat class, sex, or weight category, but was derived more substantially from technical and biomechanical indicators, whereas the linear model appeared to rely relatively more on the grouping covariates.

**Figure 5:**
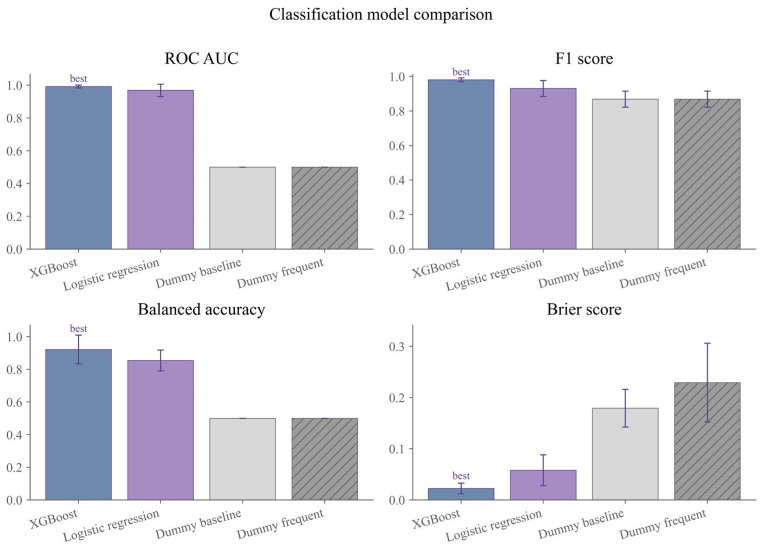
Comparison of discriminative performance across secondary classification models.

In the secondary classification analysis, all formal models substantially outperformed the baseline model (Figure 5). Grouped cross validation showed that XGBoost Classifier achieved the best classification performance, with an ROC AUC of 0.992, an F1 score of 0.981, a balanced accuracy of 0.922, and a Brier score of 0.022, whereas Logistic Regression yielded an ROC AUC of 0.968, an F1 score of 0.930, a balanced accuracy of 0.854, and a Brier score of 0.058. By comparison, the baseline model produced an ROC AUC of 0.500 and a balanced accuracy of 0.500, indicating that the formal classification models performed substantially better than random or majority-class discrimination.

**Figure 6:**
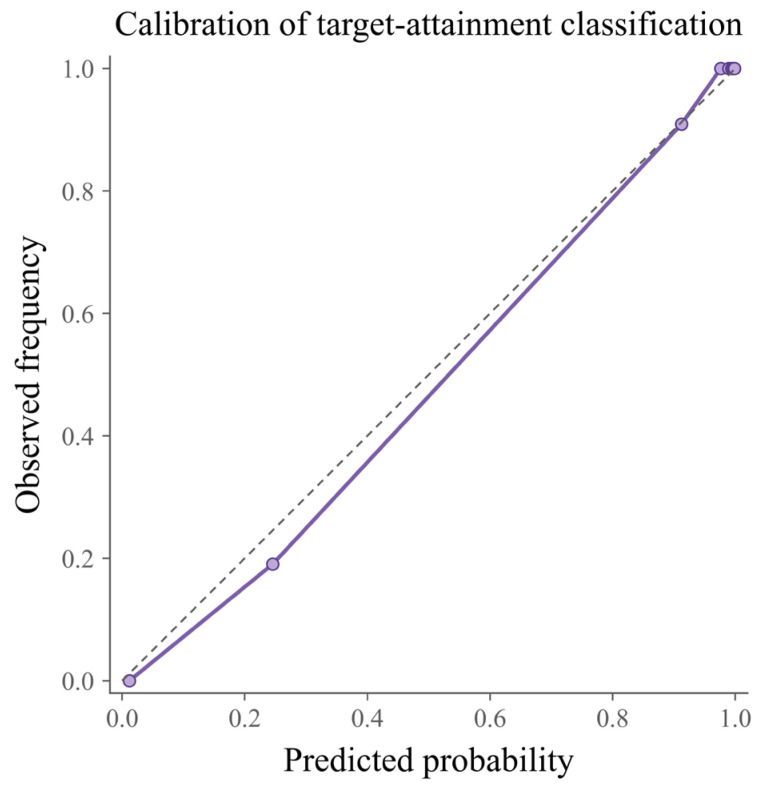
Calibration performance of the best classification model. The purple solid line with circular markers represents the calibration curve of the best-performing classifier, and the dashed diagonal line indicates perfect calibration, where predicted probabilities equal observed frequencies.

Additional threshold-based robustness analyses indicated that classification performance remained generally stable across different target attainment thresholds. When the threshold was set at 0%, 1%, and 2%, the proportions of positive cases were 76.16%, 84.88%, and 89.53%, respectively. Under these conditions, XGBoost Classifier achieved ROC AUC values of 0.992, 0.973, and 0.996, with corresponding balanced accuracy values of 0.922, 0.965, and 0.960, whereas Logistic Regression yielded ROC AUC values of 0.968, 0.962, and 0.996. Calibration analysis further showed good agreement between predicted probabilities and observed event frequencies for the best-performing classifier, indicating satisfactory probability calibration (Figure 6). Nevertheless, because the secondary classification outcome was derived by thresholding the primary regression label, these findings should be regarded as supportive rather than primary evidence and should not displace the central role of the primary regression analysis in this study.

## 4. Discussion

### 4.1. Main Findings

Despite the growing use of sensor-based and instrumented monitoring in rowing, existing studies have focused predominantly on descriptive biomechanical assessment or on linear associations between isolated indicators and performance, whereas less evidence is available on how multi-source monitoring data can be integrated into an interpretable framework for performance evaluation and training decision support. This gap is important in elite rowing because coaches must make timely judgments not only about whether performance is improving, but also about how far an athlete remains from the predefined target and which technical domains should be prioritized in subsequent training. The rationale for the present study was therefore that the practical challenge in high-performance rowing is no longer data acquisition alone, but the translation of complex monitoring outputs into structured, interpretable, and actionable feedback. On this basis, the primary purpose of this study was to construct a unified training monitoring dataset from real-world sensor-derived biomechanical measurements, develop models to predict performance gaps relative to target 2 km performance, and identify the key training monitoring indicators associated with those gaps.

The main findings can be summarized as follows: First, all formal models outperformed their respective baseline models, indicating that routine training monitoring variables contained meaningful predictive information regarding athletes’ performance gaps relative to the target. Within the primary regression task, XGBoost Regressor performed best in terms of absolute error control, whereas Elastic Net provided complementary value through stronger linear interpretability and favorable RMSE and R^2^ performance. Second, after removal of grouping variables such as boat class, sex, and weight category, the predictive performance of best MAE model remained largely stable, suggesting that the primary predictive signal was derived mainly from measured technical and biomechanical features rather than from background event labels. Third, across both global feature importance and sparse linear coefficients, mean boat velocity, minimum boat velocity, stroke rate, distance per stroke, boat efficiency, oar-blade efficiency, and several angular technical indicators consistently emerged as important variables, suggesting that performance gaps relative to the target were more likely to reflect the coupled alignment of boat-speed maintenance, rhythm organization, and propulsive efficiency than the influence of any single isolated metric.

Taken together, these findings suggest that the main contribution of the study lies not merely in showing that performance gaps can be predicted, but in demonstrating how routine rowing monitoring data from a single elite-team context can be reorganized into a predictive, interpretable, and feedback-oriented framework. In this sense, the study extends the value of sensor-derived rowing measurements from descriptive monitoring toward data-driven performance evaluation and decision support within the present team setting. These findings should therefore be interpreted as within-team applied evidence rather than as evidence of a broadly validated general framework.

### 4.2. Sport-Specific Significance of Key Training Monitoring Indicators

The key training monitoring indicators identified in this study were concentrated primarily in mean boat velocity, minimum boat velocity, stroke rate, distance per stroke, and measures of boat and oar-blade efficiency, and the model further suggested that athletes’ performance gaps relative to target 2 km biomechanical test performance were not simply the result of a linear accumulation of isolated indicators, but rather reflected a coupled mismatch among boat-speed organization, rhythm control, and propulsive efficiency [26,27,28].

The prominence of boat speed-related variables indicates that the critical determinant of performance gaps lies not only in whether a greater speed can be attained, but also in whether a more stable speed structure can be maintained throughout the stroke cycle, that is, in the overall quality of speed organization [29]. Fluctuations in boat velocity within the stroke cycle increase additional hydrodynamic resistance and ultimately translate into objective losses in performance time [30,31]. In the present models, minimum boat velocity often occurred in the early phase of the drive, whereas greater mean boat velocity was associated not only with greater stroke rate but also with the ability to sustain force application over a greater proportion of the stroke cycle [32,33]. The fact that both minimum and mean boat velocity were retained among the key indicators suggests that the ability to maintain speed and control intra cycle speed fluctuation may be more closely related to the essence of rowing performance than peak speed alone. From a practical perspective, this finding implies that pacing strategy training should not focus exclusively on maximizing peak speed capacity, but should also emphasize whether effective propulsion can be sustained throughout the stroke cycle with smaller losses in boat speed.

The importance of stroke rate and rhythm-related variables further supports the view that performance gaps are not merely a matter of power output, but depend critically on the athlete’s ability to regulate rhythm. Previous work has shown that stroke rate and rowing power output jointly mediate the relationship between multiple technical variables and boat speed, such that many biomechanical features acquire meaningful interpretive value only when considered under given stroke rate and output conditions [3,34]. In the present study, the importance of stroke rate does not imply that a greater stroke rate is itself the sole objective; rather, stroke rate should be understood as a mediating variable linking technical execution to propulsive outcome, thereby underscoring the athlete’s capacity to organize propulsion rhythm, distribute movement timing, and coordinate output patterns under a given task demand.

The simultaneous inclusion of distance per stroke, boat efficiency, and oar-blade efficiency among the key variables further indicates that the model captured the quality of propulsion transfer rather than power output alone [28,29]. Although variables such as stroke rate and distance per stroke were directionally associated with more favorable target-relative performance, several biomechanical indicators related to angle and timing still retained clear technical significance under comparable output and stroke rate conditions. In other words, rowing performance cannot be reduced simply to producing more output, but depends more fundamentally on how effectively that output is converted into forward boat motion. Athletes’ performance gaps relative to the target are therefore not attributable solely to insufficient output; more often, they reflect excessive loss during the conversion of output into propulsion. In practical training settings, whether each stroke effectively translates output into forward progression is precisely one of the core concerns of coaching evaluation.

The shared significance of these key training monitoring indicators lies not in merely reaffirming that certain single variables are associated with rowing performance, but in suggesting that performance gaps in rowing should be understood more appropriately as a coupled problem jointly shaped by boat speed organization, rhythm regulation, and propulsion transfer. It is at this level that the interpretive results of the present models acquire practical value, because they condense a set of previously parallel and fragmented testing outputs into a more mechanism-oriented explanatory framework, thereby providing a biomechanical basis for identifying performance limitations and prioritizing intervention targets in subsequent training [35].

### 4.3. Translational Value of Sensor-Derived Monitoring for Training Decision Support

From the perspective of sensor applications, the present study is not centered on proposing a new hardware device, but on enhancing the interpretive and practical value of sensor-generated monitoring outputs in an elite sports setting. Although the present study does not introduce a novel sensing device, it advances the application value of existing sensor-based rowing monitoring systems by improving the integration, interpretation, and practical usability of the data they generate. The main contribution of the study is to transform routine sensor- and instrumentation-derived rowing measurements from isolated descriptive outputs into an integrated and interpretable decision-support framework. A fundamental challenge in current high-performance rowing practice lies in the following mismatch: although routine testing and boat-based instrumentation can now generate large volumes of technical and biomechanical information, coaches in real-world training environments still face difficulties related to monitoring complexity, limited interpretability, and the lack of alignment between short term judgment and long-term evaluation [34,35]. Accordingly, the central challenge in training monitoring is no longer data acquisition alone, but rather how information from different sources can be transformed into an integrated basis for training adjustment and evaluation of training effects.

From a practical training perspective, the decision support framework developed in this study translates originally fragmented testing outputs into a unified evaluative coordinate centered on performance gaps relative to target 2 km performance. In this way, monitoring no longer remains at the level of isolated description of individual tests, but can instead directly inform the more practically relevant question of how far the athlete currently remains from the predefined target. In rowing, the widespread application of sensor technologies has already made it possible to measure kinematic, biomechanical, and technical variables with reasonable stability, yet the practical limitation has been the absence of a framework capable of organizing these variables into results that can support timely judgment [4,36]. The present framework addresses this limitation by shifting from parallel presentation of multiple indicators to the identification of meaningful combinations of key indicators. The repeated emergence of mean boat velocity, minimum boat velocity, stroke rate, distance per stroke and efficiency-related variables across models indicates that the approach does not simply accumulate variables, but instead helps coaches identify which indicators are most worthy of priority interpretation and intervention.

More importantly, the practical value of the present study does not lie in replacing coaches with algorithms, but in providing a more stable data-based foundation for prioritization in training decision making [37]. In elite rowing, the central issue is often not whether coaches know which indicators matter, but which category of problem should be addressed first within limited training time. The models developed in this study suggest that performance gaps are more appropriately understood as a coupled problem involving boat speed organization, rhythm regulation, and propulsion transfer, thereby helping coaches judge whether current limitations are more likely to arise from insufficient speed maintenance, disordered rhythm organization, or reduced propulsive efficiency [38]. Compared with traditional experience-based judgment, this interpretive framework does not alter the central role of coaching expertise, but it can substantially improve the structure and repeatability of training feedback. This orientation is consistent with current developments in explainable artificial intelligence in sport science, where the overall volume of AI research remains limited and much of the existing work has focused on technical demonstration rather than user-oriented practical application, thereby constraining coaches’ and analysts’ ability to trust, validate and apply model outputs [13]. In the present study, primary model performance, sparse linear feature signatures, global importance rankings, and the persistence of the main predictive signal after removal of grouping variables together formed an interpretive framework that was more closely aligned with the needs of real training settings, helping to shift machine learning from a purely predictive tool toward a tool for training decision support [39].

### 4.4. Limitation

The present study has several limitations that should be considered. First, the data were obtained from a single elite national rowing team and, although they were collected in a real-world training setting and therefore carry clear practical relevance, the external generalizability of the models remains to be further examined in larger samples and in teams of different competitive levels. Second, although grouped internal validation, limited predefined hyperparameter comparison, leakage control, baseline model comparison, and sensitivity analyses were used to reduce methodological optimism, the effective modeling sample for the primary task remained modest relative to the dimensionality of the candidate predictor space. Accordingly, the reported model performance may still overestimate stability to some extent and should be interpreted as preliminary within-team evidence rather than definitive evidence of stable generalizable performance. Third, the study relied on exported sensor-derived biomechanical monitoring outputs and aggregated analytical indicators generated within a routine team-monitoring workflow, rather than on raw continuous sensor streams modeled directly from first acquisition. As a result, the analytical dataset may still be influenced by factors such as measurement uncertainty, calibration variability, operator dependence, and system-specific processing logic, which could not be fully quantified within the present retrospective design. In addition, the modeling framework was based primarily on aggregated biomechanical monitoring indicators and did not yet incorporate more raw continuous time-series signals or multimodal information such as video data. Consequently, further work is needed to more fully elucidate the mechanisms underlying sport-specific performance formation.

## 5. Conclusions

Using biomechanical measurements collected during the 2024–2025 training seasons of the Chinese National Rowing Team, this study established a unified training monitoring dataset and developed models to predict athletes’ performance gaps relative to target 2 km performance. Models based on routine training-monitoring variables were able to characterize, with encouraging within-team consistency under grouped internal validation, the gap between athletes’ current condition and the target performance level. XGBoost Regressor achieved the best performance in terms of absolute error control, whereas Elastic Net showed greater advantages in overall model stability and interpretability. Model interpretation and robustness analyses further indicated that mean boat velocity, minimum boat velocity, stroke rate, distance per stroke, and efficiency-related indicators were the core variables associated with performance gaps and that the primary predictive signal arose mainly from technical and biomechanical indicators rather than from background grouping information such as boat class, sex, or weight category. Overall, these findings indicate that, within the present elite-team setting, rowing biomechanical measurements derived from routine sensor-based monitoring can be extended beyond quantitative description to support interpretable prediction of performance gaps and identification of key training indicators. In this respect, the study illustrates the team-specific applied value of sensor-generated monitoring outputs for data-driven performance evaluation and training decision support, while broader validation in other teams and monitoring environments remains necessary.

## Figures and Tables

**Table 1:** Core training monitoring indicators jointly identified by Elastic Net and LASSO.

Feature	Elastic Net Coefficient	Direction	LASSO Coefficient	Direction	Domain
Max boat speed (m/s)	5.9968	positive	6.7785	positive	Boat speed performance
Stroke rate (spm)	4.9461	positive	4.9585	positive	Rhythm control
Boat power (w)	−3.1791	negative	−3.2988	negative	Power output
Distance per stroke (m)	3.0460	positive	3.2328	positive	Propulsive efficiency
Min boat speed (m/s)	−2.6979	negative	−3.1367	negative	Boat speed maintenance
Average boat speed (m/s)	2.9190	positive	2.9812	positive	Boat speed maintenance
Boat efficiency (%)	2.8544	positive	2.8752	positive	Propulsive efficiency

Note: Values shown are the core training-monitoring indicators jointly retained by the Elastic Net and LASSO models. The coefficient direction indicates the association direction with the relative 2 km time gap. A positive coefficient indicates that a greater value of the corresponding variable is associated with a larger relative time gap, whereas a negative coefficient indicates that a greater value is associated with a smaller relative time gap. Coefficient magnitude is intended for within-model comparison only and should not be interpreted as a causal effect or a direct estimate of practical effect size.

## Data Availability

The de-identified analytical dataset used in this study, together with a data dictionary and supporting documentation, is provided in the Supplementary Materials. The shared files correspond to the dataset used for the manuscript-level analyses. Raw source monitoring files are not openly shared because they contain protected athlete information and operational metadata from routine elite-team monitoring.

## Supplementary Materials

The following supporting information can be downloaded at: https://www.mdpi.com/article/10.3390/s26103006/s1, Dataset S1: De-identified analytical dataset used for the manuscript-level analyses; File S1: Supporting documentation describing dataset construction, variable definitions, matching procedures, feature categories, model input variables, and de-identification procedures.
